# Construction and validation of a predictive model for poor long-term prognosis in severe acute ischemic stroke after endovascular treatment based on LASSO regression

**DOI:** 10.3389/fneur.2025.1535679

**Published:** 2025-04-14

**Authors:** Yingli Zhang, Yan Guo, Zhenpeng Zhang, Jie Han

**Affiliations:** ^1^The First Affiliated Hospital of Dalian Medical University, Dalian, China; ^2^Panjin Central Hospital, Panjin, China; ^3^Shanghai Medical College, Fudan University, Shanghai, China

**Keywords:** severe acute ischemic stroke, endovascular treatment, poor prognosis, predictive model, clinical application potential

## Abstract

**Objective:**

We aimed at establishing a predictive model for poor long-term prognosis (3 months post-treatment) following endovascular treatment (EVT) for severe acute ischemic stroke (AIS) and evaluating its predictive performance.

**Methods:**

The patients with severe AIS (NIHSS score ≥ 16) who received EVT were divided into a modeling group (178 patients), an internal validation group (76 patients), and an external validation group (193 patients). Internal and external validation were performed using cross-validation. Poor long-term prognosis was defined as a modified Rankin Scale (mRS) score > 2 at 3 months after the stroke. Univariate analysis and LASSO regression were used to select risk factors, and a logistic regression model was established to create a nomogram. The model’s performance and clinical applicability were evaluated using the area under the receiver operating characteristic (ROC) curve (AUC), calibration curves, and decision curves.

**Results:**

Five predictive factors were identified: baseline NIHSS score (OR = 1.096, 95% CI: 1.013–1.196, *p* = 0.0279), symptomatic intracranial hemorrhage (OR = 6.912, 95% CI: 1.758–46.902, *p* = 0.0156), time from puncture to reperfusion (OR = 1.015, 95% CI: 1.003–1.028, *p* = 0.0158), age (OR = 1.037, 95% CI: 1.002–1.076, *p* = 0.0412), which were found to be risk factors for poor long-term prognosis after EVT for severe AIS. Collateral circulation was identified as a protective factor (OR = 0.629, 95% CI: 0.508–0.869, *p* = 0.0055). Based on these five factors, a nomogram was constructed to predict poor long-term prognosis after EVT. The ROC curve showed that the AUC for predicting poor long-term prognosis was 0.7886 (95% CI: 0.7225–0.8546) in the modeling group, 0.8337 (95% CI: 0.7425–0.9249) in the internal validation group, and 0.8357 (95% CI: 0.7793–0.8921) in the external validation group. The calibration curve and clinical decision curve demonstrated good consistency and clinical utility of the model.

**Conclusion:**

The predictive model for poor long-term prognosis following EVT for severe AIS has accurate predictive value and clinical application potential.

## 1 Introduction

Severe acute ischemic stroke (AIS) is characterized by a sudden onset, rapid progression, and high severity, often leading to significant disability and mortality, imposing a substantial burden on patients’ families. Currently, endovascular treatment (EVT) is the frontline therapeutic strategy for patients with severe AIS (1). This approach is essential for timely vascular recanalization, restoration of blood flow to the infarcted area, and mitigation of brain tissue damage. However, despite successful recanalization of the occluded vessels, nearly half of these patients experience poor functional outcomes within 90 days post-stroke onset (2, 3). Patients were assigned to the favorable outcome group (90-day mRS ≤2) and the poor outcome group (90-day mRS >2). Identifying the factors that influence these functional outcomes is therefore crucial for improving prognosis. Due to the acute onset, severe condition, and high mortality of these patients, conducting clinical research in this population is extremely challenging. As a result, studies on this group remain limited (4, 5). The existing studies are predictive models generally limited to anterior or posterior circulation cases (6, 7). Endovascular treatment is currently the most effective approach for these patients; however, no systematic studies or comprehensive data are available to assess its benefit rate.

Several previous studies have analyzed clinical factors influencing prognosis, including age, NIHSS score, and symptomatic hemorrhage. However, these findings have often been limited to logistic regression with moderate predictive power (8–11). Although factors affecting the prognosis of endovascular treatment for acute severe ischemic stroke have been explored, no predictive models specifically targeting long-term outcomes in these patients have been developed. In recent years, various machine learning algorithms have been applied in clinical research, such as decision tree algorithms, support vector machines (SVM), linear discriminant analysis (LDA), and k-nearest neighbors (KNN) (12, 13). Advancements in machine learning and deep learning technologies have significantly improved the performance of various predictive models, highlighting the need for a dedicated model to predict long-term outcomes in this critical patient population.

Previous studies have explored various factors influencing EVT outcomes in severe AIS but have yet to establish a predictive model applicable to clinical practice. In this study we analyzed clinical data using the Least Absolute Shrinkage and Selection Operator (LASSO) (14) regression to identify valuable predictors and established a predictive model for long-term poor prognosis following EVT in severe AIS. LASSO regression effectively handles multicollinearity and prevents overfitting. By employing this technique, we identified the most predictive variables from a broad range of potential risk factors, significantly enhancing the model’s precision and predictive power, which have been well-documented across various medical research fields. The model underwent both internal and external validation, providing new insights for early diagnosis of poor long-term outcomes in this patient population. Such a model would improve prognostic assessments and targeted clinical decisions. Additionally, it would encourage practitioners to enhance thrombectomy techniques and streamline treatment processes.

## 2 Subjects and methods

### 2.1 Study subjects

We collected data from 254 patients with severe AIS who received EVT at the First Affiliated Hospital of Dalian Medical University from January 1, 2019, to January 1, 2024. Using the R caret package, patients were randomly divided into a modeling group (*n* = 178) and an internal validation group (*n* = 76) at a 7:3 ratio to develop and validate the model internally. Additionally, data from 193 patients who received EVT for severe AIS at Panjin Central Hospital during the same period were collected as an external validation cohort.

### 2.2 Inclusion and exclusion criteria

Inclusion criteria: (1) age ≥ 18 years; (2) National Institutes of Health Stroke Scale (NIHSS) score ≥ 16; (3) pre-stroke modified Rankin Scale (mRS) score < 2; and (4) onset-to-bridging thrombectomy time < 4.5 h or direct EVT within 24 h of onset. Exclusion criteria: based on exclusion criteria for intravenous thrombolysis (2).

### 2.3 Methods

#### 2.3.1 Data collection

Data were collected on demographics (including gender and age), medical history (including history of stroke, diabetes, hypertension, hyperlipidemia, atrial fibrillation, coronary artery disease, smoking, and alcohol use). The data from different cohorts are presented in Tables 1, 2.

**Table 1:** Baseline characteristics of subjects in the modeling and internal validation groups.

Variable	Modeling group (*n* = 178)	Internal validation group (*n* = 76)	*t*/*χ*^2^/*Z* value	*p* value
Sample size	178	76		
Age (years, median [IQR])	68 (61–76)	68.5 (61–73.75)	−0.893	0.372
Male (%)	130 (73.0%)	57 (75.0%)	0.106	0.745
Preoperative NIHSS Score (median [IQR])	22 (20–25)	21.5 (18.25–24)	−1.321	0.187
Systolic BP (mmHg, χ ± s)	151.58 ± 23.622	156.37 ± 24.906	0.972	0.080
Diastolic BP (mmHg, χ ± s)	88.33 ± 13.358	90.86 ± 15.216	−1.325	0.187
Hypertension	104 (58.4%)	50 (65.8%)	1.209	0.271
Diabetes	44 (24.7%)	19 (25.0%)	0.002	0.962
Atrial Fibrillation	70 (39.3%)	25 (32.9%)	0.941	0.332
Coronary Artery Disease	25 (14.0%)	14 (18.4%)	0.785	0.376
History of Cerebrovascular Disease	20 (11.2%)	13 (17.1%)	1.623	0.203
Smoking History	38 (21.3%)	23 (30.3%)	2.320	0.128
Alcohol Use	29 (16.3%)	15 (19.7%)	0.441	0.507
Occlusion Site (%)				
Anterior Circulation	157 (88.2%)	68 (89.5%)	0.085	0.770
Posterior Circulation	21 (11.8%)	8 (10.5%)	0.085	0.770
Surgical Information (%)				
Intra-arterial Thrombolysis	5 (2.8%)	1 (1.3%)	0.515	0.473
Stent Retrieval	169 (94.9%)	76 (100%)	3.984	0.046^*^
Balloon Angioplasty	50 (28.1%)	18 (23.7%)	0.527	0.468
Permanent Intracranial Stenting	57 (32.0%)	17 (22.4%)	2.404	0.121
Onset-to-Hospital Time (min, median [IQR])	180 (120–270)	180 (120–240)	−0.359	0.719
Puncture-to-Recanalization Time (min, median [IQR])	75 (50–98.5)	80 (58.5–105)	−1.097	0.273
Onset-to-Recanalization Time (min, median [IQR])	336 (268.75–422.5)	332.5 (260–392.5)	−0.490	0.624
Thrombectomy Attempts (≥3)	38 (21.3%)	19 (25.0%)	0.408	0.523
Preoperative Thrombolysis	36 (20.2%)	13 (17.1%)	0.333	0.564
Collateral Circulation (median [IQR])	1 (0–2)	1 (0–2)	−1.164	0.244
Etiology (%)				
Large-artery Atherosclerosis	95 (53.4%)	43 (56.6%)	0.221	0.638
Embolic	75 (42.1%)	28 (36.8%)	0.619	0.431
Other Causes	8 (4.5%)	5 (6.6%)	0.477	0.490
Complications (%)				
Symptomatic Hemorrhage	27 (15.2%)	8 (10.5%)	0.966	0.320

* Statistical significance at *p* < 0.05.

**Table 2:** Univariate analysis of variables for unfavorable prognosis in the modeling Group.

Variable	Favorable prognosis group	Unfavorable prognosis group	*t*/*χ*^2^/*Z* value	*p* value
Sample Size (n)	71	107		
Age (years, χ̅±s)	65.72 ± 10.374	69.78 ± 9.696	−2.648	0.009
Male (n, %)	54 (78.3%)	76 (69.7%)	1.563	0.211
Baseline NIHSS Score (median [IQR])	20 (18–24)	22 (20–26)	−2.985	0.003
Systolic BP (mmHg, χ̅±s)	145.58 ± 21.483	154.73 ± 24.087	−2.574	0.011
Diastolic BP (mmHg, χ̅±s)	84.49 ± 11.698	89.97 ± 13.179	−2.821	0.005
History (n, %)				
Hypertension	35 (50.7%)	69 (63.3%)	2.753	0.097
Diabetes	11 (15.9%)	33 (30.3%)	4.665	0.031
Atrial Fibrillation	25 (36.2%)	45 (41.3%)	0.452	0.501
Coronary Artery Disease	7 (10.1%)	18 (16.5%)	1.420	0.233
Cerebrovascular Disease History	7 (10.1%)	13 (11.9%)	0.134	0.714
Smoking	15 (21.7%)	23 (21.1%)	0.010	0.919
Alcohol Use	7 (10.1%)	22 (20.2%)	3.122	0.077
Occlusion Site (n, %)				
Anterior Circulation	66 (93%)	92 (86%)	2.083	0.149
Posterior Circulation	5 (7%)	15 (14%)	2.083	0.149
Endovascular Therapy (n, %)				
Arterial Thrombolysis (Fisher)	3 (4.3%)	2 (1.8%)	0.129	0.294
Stent Retrieval	65 (94.2%)	104 (95.4%)	0.045	0.720
Balloon Angioplasty	20 (29%)	30 (27.5%)	0.394	0.832
Permanent Intracranial Stent	24 (34.8%)	33 (30.3%)	−1.564	0.530
Onset to Hospital Time (min, median [IQR])	210 (135–300)	180 (120–240)	−2.302	0.118
Puncture to Recanalization Time (min, median [IQR])	70 (50–85)	85 (55–107.5)	−0.632	0.021
Onset to Recanalization Time (min, median [IQR])	330 (255–420.5)	340 (270–425)	−0.490	0.528
Thrombectomy Attempts ≥3 (n, %)	15 (21.7%)	23 (21.1%)	0.010	0.919
Pre-procedure Thrombolysis (n, %)	17 (24.6%)	19 (17.4%)	1.360	0.244
Collateral Circulation (median [IQR])	2 (0–2)	0 (0–1)	−4,526	0.000
Etiology (n, %)				
Large Artery Atherosclerosis	38 (55.1%)	57 (52.3%)	0.131	0.717
Embolism	28 (40.6%)	47 (43.1%)	0.112	0.738
Other Causes	3 (4.3%)	5 (4.6%)	0.006	0.940
Complications (n, %)				
Symptomatic Hemorrhage	2 (2.9%)	25 (22.9%)	13.183	0.000

#### 2.3.2 Treatment

For patients eligible for intravenous thrombolysis, 0.9 mg/kg alteplase was administered intravenously after excluding contraindications, followed by bridging to EVT. Femoral artery access was used for all procedures, and the choice of anesthesia (local or general) depended on the patient’s condition. The primary treatment strategies included stent retrieval, thrombus aspiration, or a combination of both. If the residual stenosis rate at the previously occluded site was ≥70% or if distal thrombus migration occurred, rescue therapies such as balloon angioplasty, stent placement, or intra-arterial thrombolysis were performed.

#### 2.3.3 Assessment

The following assessments were recorded: baseline NIHSS score, collateral circulation (classified as poor with preoperative DSA scores of 0–2 and good with scores of 3–4), modified Thrombolysis in Cerebral Infarction (mTICI) grade (with scores of 0–2a indicating unsuccessful recanalization and 2b–3 indicating successful recanalization), Patients were assigned to the long-term favorable outcome group (mRS ≤2 at 90 days) and the long-term unfavorable outcome group (mRS >2 at 90 days). Two associate chief neurologists independently and blindly reviewed imaging data; any discrepancies were resolved through consultation.

### 2.4 Statistical analysis

For model development, prior to performing LASSO regression, we first conducted univariate analysis to select potential variables. This step was based on prior studies that have successfully used univariate analysis for variable selection, which demonstrated good model accuracy and clinical applicability (15). A univariate analysis was conducted on 178 subjects based on poor prognosis outcomes. Variables with a *p*-value <0.05 were considered statistically significant. These significant variables from the univariate analysis were further processed using LASSO regression “glmnet” package in R. The optimal *λ* was determined using 10-fold cross-validation. The coefficient profile plot illustrates the relationship between log(*λ*) and the regression coefficients, where increasing log(*λ*) results in the gradual shrinkage of coefficients, with some approaching zero. The cross-validation curve presents the mean squared error (MSE) across different *λ* values. The left dashed line represents the λ corresponding to the minimum MSE (NULL), while the right dashed line indicates the λ value one standard error above the minimum MSE (0.04739061). The final model was selected based on this λ value, ensuring a balance between model complexity and predictive accuracy, which identified non-zero features deemed relevant. These variables were then included in a multivariate logistic regression model to identify the final prognostic factors, which were subsequently used to construct the nomogram.

Based on the significant predictors identified, a nomogram prediction model was constructed using the “rms” package in R 4.4.1. In this model, each predictor was assigned a score according to its contribution to the overall prediction, with higher scores corresponding to a greater probability of poor prognosis. The total score, obtained by summing the individual predictor scores, was then converted into a probability of poor prognosis, allowing for a straightforward and visualized risk assessment. For example, the nomogram assigns a score to each predictive factor within a range of 0 to 100 points. The cumulative score across all factors is then translated into the predicted probability of poor prognosis. For instance, for a 65-year-old patient with acute severe ischemic stroke, an NIHSS score of 24, a puncture-to-reperfusion time of 70 min, a collateral circulation score of 2 points, and symptomatic hemorrhage, the total score amounts to 176 points, which corresponds to an 81.5% probability of poor prognosis.

To evaluate model performance, the receiver operating characteristic (ROC) curve was generated using the “pROC” package, and the area under the curve (AUC) was calculated to assess predictive accuracy, where a value closer to 1 indicates a higher consistency between predicted and actual outcomes. Calibration was assessed using the Hosmer-Lemeshow test, performed with the “Resource Selection” package, where a *p*-value >0.05 indicates a well-calibrated model. To ensure stability, bootstrap internal validation was conducted using the “caret” package with 1,000 resampling iterations. The clinical utility of the nomogram was further assessed using decision curve analysis (DCA) via the “ggDCA” package in R 4.2.1. External validation was conducted using a cohort from a different hospital. This external validation cohort exhibited distinct clinical characteristics compared to the modeling group, reflecting variations in treatment protocols and management strategies across institutions. To validate the model, cross-validation (specifically k-fold cross-validation) was employed. This method was utilized for both internal and external validation to ensure the robustness and generalizability of the model.

Statistical analyses were conducted using SPSS 26.0 and R 4.4.1 software. For continuous data with a normal distribution, results were expressed as mean ± standard deviation (x̅ ± s), and comparisons between groups were performed using an independent sample *t*-test. For non-normally distributed continuous data, results were presented as median (Q1, Q3), and group comparisons were made using the Mann–Whitney *U* test. Categorical data were expressed as frequencies and percentages (%), with group comparisons conducted using the *χ*^2^ test or Fisher’s exact test, as appropriate. All tests were two-sided, with a significance level set at *α* = 0.05.

## 3 Results

### 3.1 Baseline characteristics

Baseline characteristics of the 178 patients in the modeling group and 76 patients in the internal validation group were compared. There was a significant difference in systolic blood pressure and stent retrieval between the two groups (*p* < 0.05). No statistically significant differences were observed between the two groups in terms of age, gender, medical history (hypertension, diabetes, atrial fibrillation, history of cerebrovascular disease, coronary artery disease, smoking, alcohol use), diastolic blood pressure at admission, baseline NIHSS score, occlusion location (anterior circulation, posterior circulation), surgical interventions (balloon angioplasty, permanent intracranial stenting), time from symptom onset to hospital arrival, puncture-to-recanalization time, onset-to-recanalization time, collateral circulation status, number of thrombectomy attempts (≥3), preoperative thrombolysis, etiological classification (large-artery atherosclerosis, embolic, other), and incidence of symptomatic hemorrhage (*p* > 0.05). Details are provided in Table 1.

### 3.2 Variable selection in the modeling group

A univariate analysis was conducted on the 178 subjects in the modeling group, dividing them into favorable prognosis and unfavorable prognosis groups. The results showed that age, baseline NIHSS score, systolic blood pressure, diastolic blood pressure, history of diabetes, puncture-to-recanalization time, collateral circulation, and symptomatic hemorrhage were significantly different between the two groups (*p* < 0.05). These results are presented in Table 2.

**Figure 1:**
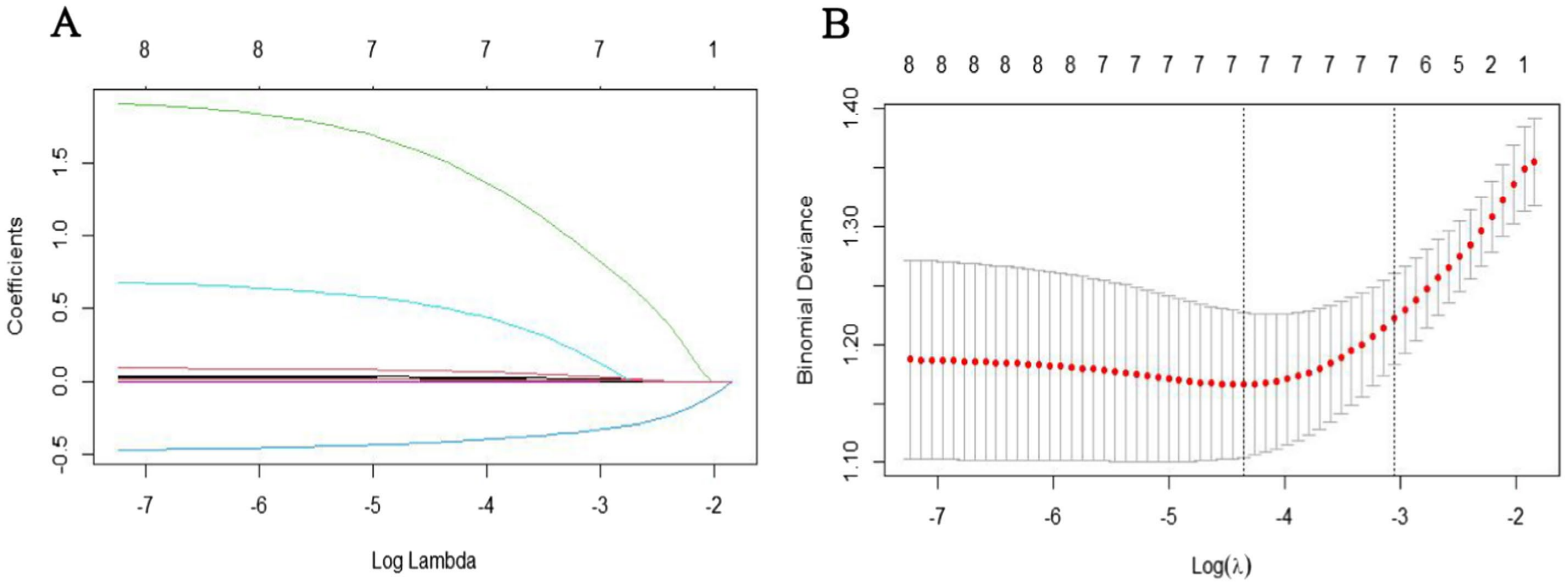
Variable selection using LASSO regression: **(A)** LASSO coefficient profiles of the candidate predictors. **(B)** Selection of the optimal penalization coefficient in LASSO regression.

To further identify the most significant predictors for prognosis, the variables with statistical significance from the univariate analysis were included in the LASSO regression analysis. The results showed that when *λ* = 0.047, five variables with non-zero coefficients were selected: age, baseline NIHSS score, puncture-to-recanalization time, collateral circulation, and symptomatic hemorrhage (Figure 1).

### 3.3 Development of the nomogram model

**Figure 2:**
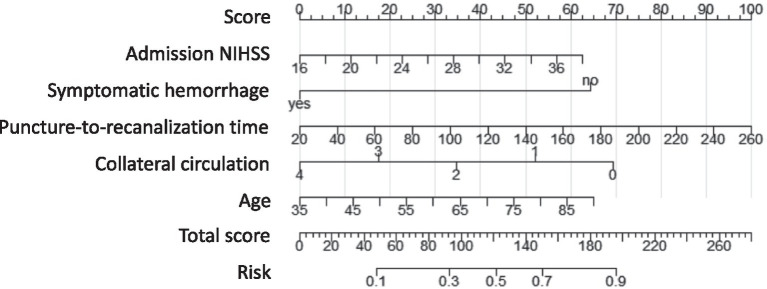
Nomogram predicting long-term poor outcomes following EVT for acute severe cerebral infarction.

The five selected factors were included in a multivariate logistic regression analysis. The results showed that age, baseline NIHSS score, puncture-to-recanalization time, and symptomatic hemorrhage were identified as risk factors for unfavorable long-term prognosis, while collateral circulation was found to be a protective factor (Table 3). Based on the five identified predictors, a nomogram model was developed to predict the long-term unfavorable prognosis of acute severe cerebral infarction following EVT. The model is illustrated in Figure 2.

**Table 3:** Multivariate logistic regression analysis for unfavorable long-term prognosis in acute severe cerebral infarction.

Variable	*B*	OR (95% CI)	*p* value
Age	0.036	1.037 (1.002–1.076)	0.041
Baseline NIHSS score	0.092	1.096 (1.013–1.196)	0.028
Puncture-to-recanalization time	0.014	1.015 (1.003–1.028)	0.016
Collateral circulation	−0.462	0.629 (0.458–0.869)	0.006
Symptomatic hemorrhage	1.933	6.912 (1.758–46.902)	0.016

### 3.4 Internal validation of the model

**Figure 3:**
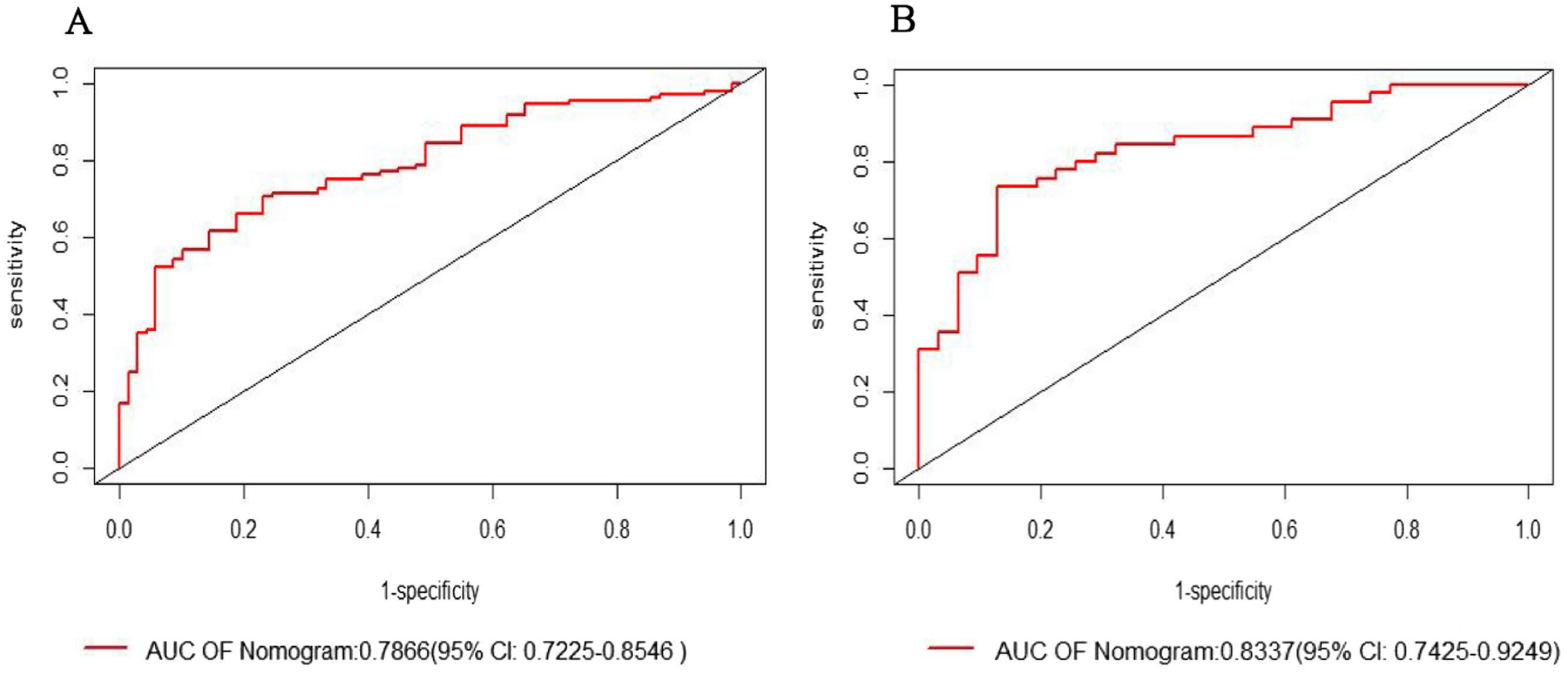
Receiver operating characteristic (ROC) curve: **(A)** Modeling group; **(B)** Internal validation group.

The ROC curve analysis revealed that the modeling group had an AUC of 0.7886 (95% CI: 0.7225–0.8546), while the internal validation group had an AUC of 0.8337 (95% CI: 0.7425–0.9249). The Youden index for the model was 0.602, with specificity and sensitivity of 76.8 and 70.6%, respectively (Figures 3A,B).

**Figure 4:**
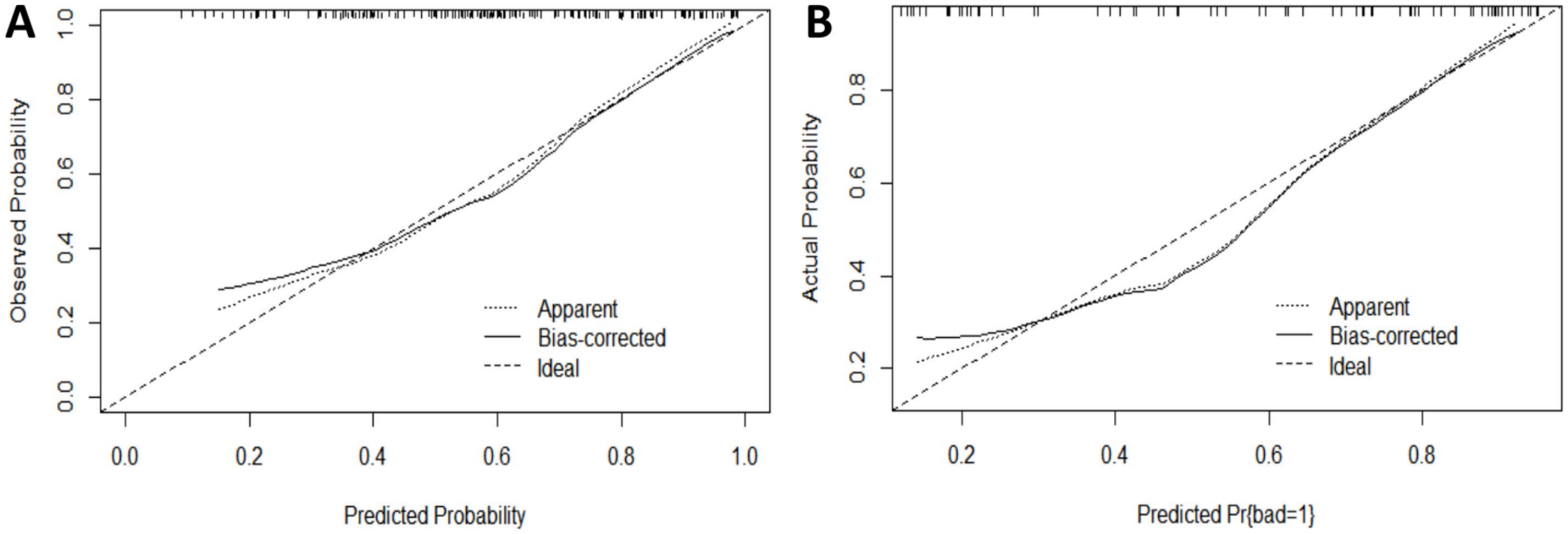
Calibration curve for the subjects: **(A)** Modeling group; **(B)** Internal validation group.

The Hosmer-Lemeshow goodness-of-fit test indicated that the modeling group had a *p*-value of 0.8769, and the internal validation group had a *p*-value of 0.9025 (*p* > 0.05). The calibration curve (Bootstrap method, *n* = 1,000) showed a close alignment between the actual and bias-corrected curves, suggesting a high degree of agreement with the observed values and indicating good calibration of the model (Figures 4A,B).

**Figure 5:**
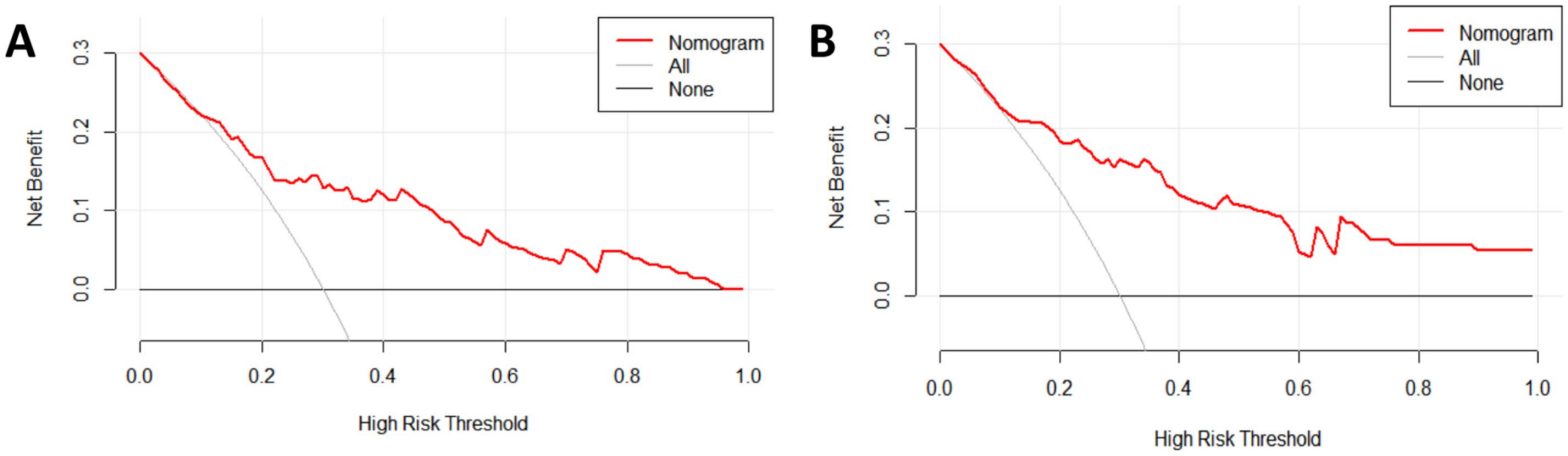
Decision curve for the subjects: **(A)** Modeling group; **(B)** Internal validation group.

The clinical utility of the nomogram model was assessed using the decision curve analysis (DCA). The results showed that the nomogram model provided greater net benefit and demonstrated high clinical applicability (Figures 5A,B).

### 3.5 External validation of the model

To further evaluate the clinical applicability of our model, we performed an external validation involving 193 patients who received EVT for severe AIS at another hospital. The comparison results showed that there were no statistically significant differences between the modeling group and the external validation group in terms of gender, medical history (hypertension, diabetes, coronary heart disease, smoking, alcohol consumption), admission systolic and diastolic blood pressure, baseline NIHSS, arterial thrombolysis, time from symptom onset to reperfusion, number of thrombectomy attempts ≥3, preoperative thrombolysis, and symptomatic hemorrhage (all *p* > 0.05). However, significant differences were found between the two groups in terms of age, history of cerebrovascular disease, atrial fibrillation, occlusion location (anterior circulation, posterior circulation), surgical procedure (stent thrombectomy, balloon dilation, permanent intracranial stenting), time from symptom onset to hospital arrival, time from puncture to reperfusion, collateral circulation, and etiology classification (large artery atherosclerosis, embolism) (all *p* < 0.05), as shown in Table 4.

**Table 4:** Comparison of clinical data between the modeling group and the external validation group.

Variable	Modeling group (*n* = 178)	External validation group (*n* = 193)	*t*/*χ*^2^/*Z* value	*p* value
Age (years, median [IQR])	68 (61–76)	66 (58–72)	−2.904	0.004
Male (n, %)	130 (73.0)	136 (70.5)	0.003	0.959
Preoperative NIHSS Score (median [IQR])	22 (20–25)	20 (16–30)	−1.491	0.136
Systolic BP (mmHg, χ̄±s)	151.58 ± 23.622	152.73 ± 22.333	0.813	0.417
Diastolic BP (mmHg, χ̄±s)	88.33 ± 13.358	89.67 ± 14.641	−0.920	0.358
Hypertension	104 (58.4)	127 (65.8)	2.144	0.143
Diabetes	44 (24.7)	47 (24.4)	0.007	0.935
Atrial Fibrillation	70 (39.3)	55 (28.5)	4.860	0.027
Coronary Heart Disease	25 (14.0)	31 (16.1)	0.294	0.588
Cerebrovascular Disease History	20 (11.2)	47 (24.4)	10.765	0.001
Smoking History	38 (21.3)	45 (23.3)	0.206	0.650
Alcohol History	29 (16.3)	31 (16.1)	0.004	0.952
Occlusion Site (n, %)				
Anterior Circulation	157 (88.2)	149 (77.2)	7.754	0.005
Posterior Circulation	21 (11.8)	44 (22.8)	7.754	0.005
Surgical Procedure (n, %)				
Arterial Thrombolysis (Fisher)	5 (2.8)	2 (1.0)	1.572	0.058
Stent Thrombectomy	169 (94.9)	152 (78.8)	20.808	0.000
Balloon Dilation	50 (28.1)	89 (46.1)	12.840	0.000
Permanent Intracranial Stent	57 (32.0)	91 (47.2)	8.838	0.003
Time from Onset to Hospital (min, median [IQR])	180 (120–270)	120 (60–180)	−7.358	0.000
Time from Puncture to Reperfusion (min, median [IQR])	75 (50–98.5)	60 (37–96)	−3.089	0.002
Time from Onset to Reperfusion (min, median [IQR])	336 (268.75–422.5)	310 (231.5–403)	−1.951	0.051
Number of Thrombectomy Attempts ≥3	38 (21.3)	30 (15.5)	2.082	0.149
Preoperative Thrombolysis	36 (20.2)	41 (21.2)	0.809	0.000
Collateral Circulation (n, %)	1 (0–2)	1 (1–2)	−4.603	0.000
Etiology Classification (n, %)				
Large Artery Atherosclerosis	95 (53.4)	126 (65.3)	5.458	0.019
Embolism	75 (42.1)	55 (28.5)	7.566	0.006
Other Causes	8 (4.5)	12 (6.2)	0.539	0.463
Complications (n, %)				
Symptomatic Hemorrhage	27 (15.2)	26 (13.5)	0.218	0.641

**Figure 6:**
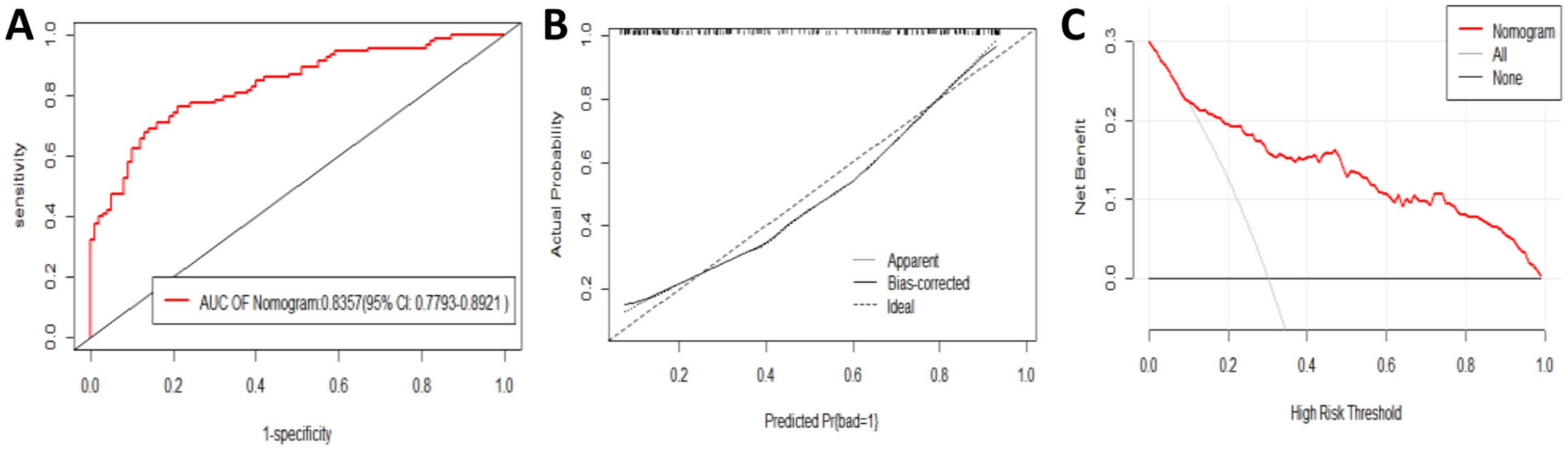
Evaluation and validation of the nomogram model in the external validation group. **(A)** Receiver operating characteristic (ROC) curve. **(B)** Calibration curve. **(C)** Decision curve.

The ROC curve analysis showed that the AUC for the external validation group was 0.8357 (95% CI: 0.7793–0.8921). The goodness-of-fit test showed a *p*-value of 0.08556 (*p* > 0.05), indicating no significant deviation. The calibration curve also demonstrated that the model’s performance closely aligned with the observed data. The decision curve analysis (DCA) revealed a threshold range of 0.1 to 1 (Figures 6A–C).

## 4 Discussion

This study developed a predictive model with significant clinical utility for aiding decisions on treatment selection, prognosis management, and rehabilitation planning. The model functions as a visualization and scoring system that combines key variables to generate a continuous score, providing an accurate risk probability for clinical events in individual patients. This score offers a simple and intuitive way for physicians to assess patient risk, enabling them to identify high-risk patients and tailor personalized treatment plans and targeted medical decisions (16). The higher the probability of poor prognosis, the greater the risk of paralysis or sudden death. In clinical practice, this would prompt physicians to focus on preventing complications and enhancing rehabilitation during the recovery period. Additionally, the model serves as a valuable communication tool, facilitating discussions between physicians, patients, and their families regarding disease risks and prognosis. Several existing predictive models evaluate outcomes following endovascular treatment (17–19), but most are limited to anterior circulation strokes and lack stratification of patient groups. In our study, we focused on acute severe ischemic stroke patients involving both anterior and posterior circulation. By incorporating factors closely associated with poor outcomes at 3 months post-endovascular treatment, we identified five key predictors—age, baseline NIHSS score, puncture-to-reperfusion time, collateral circulation, and symptomatic hemorrhage—through LASSO regression to construct a comprehensive predictive model.

Our model demonstrated good discriminatory ability (AUC: 0.7886, 95% CI: 0.7225–0.8546) and calibration (Hosmer-Lemeshow test, *p* = 0.8769). Internal validation indicated strong model consistency (AUC: 0.8337, 95% CI: 0.7425–0.9249), highlighting its robust predictive capacity, while other models typically show moderate predictive power (8–11). Unlike most previous studies (20, 21), this research further validated the model externally at another hospital. The external validation cohort, drawn from a different hospital, featured distinct clinical characteristics compared to the modeling group, representing variability in treatment protocols and management strategies across institutions. Despite this heterogeneity, the model achieved a predictive accuracy of 82.6% in the external validation group, while calibration curves and decision curve analysis (DCA) further confirmed its applicability and generalizability.

Traditional logistic regression for variable selection often encounters issues such as multicollinearity and overfitting, leading to reduced accuracy and limited clinical applicability. In contrast, LASSO regression offers superior predictive power, rigorous variable selection, and strong model fitting, making it particularly advantageous in handling multicollinearity and preventing overfitting (22). The adaptability and effectiveness of LASSO regression are well-documented across various medical research fields (23–26), underscoring its utility in enhancing predictive modeling. By utilizing LASSO regression, we significantly reduced clinical costs while efficiently identifying the most predictive variables from a large pool of potential risk factors. This approach enhances the precision and predictive capacity of our model, thereby improving its practical value in clinical decision-making.

Consistent with most predictive models, baseline NIHSS score and age are the most predictive factors for long-term unfavorable outcomes following endovascular treatment (EVT) (27). The NIHSS score, widely used for assessing neurological function in acute ischemic stroke patients, reflects the severity of neurological damage. A higher NIHSS score indicates a greater likelihood of poor prognosis after EVT. In this study, we included severe cases with NIHSS scores ≥16, and the rate of long-term poor prognosis at 3 months was found to be 50–60%, aligning with our results. Previous research has demonstrated that high NIHSS scores and severe ischemic stroke are independent risk factors for poor functional outcomes at 90 days post-EVT, which is consistent with our findings.

As age increases, the incidence of stroke doubles every decade. Stroke patients often present with comorbidities such as atrial fibrillation, coronary heart disease, hypertension, and diabetes. Advanced age is associated with poorer cerebrovascular and systemic conditions (28), resulting in more significant neurological damage at onset and severe clinical symptoms. Older patients are also more prone to symptomatic hemorrhage after EVT, face greater challenges in neurological recovery during rehabilitation, and have reduced physical resilience, all contributing to worse long-term outcomes. Previous studies have consistently identified age as an independent risk factor for predicting long-term unfavorable outcomes after EVT (29).

Symptomatic hemorrhage, one of the most important complications after endovascular treatment, has been widely studied, with many studies indicating that symptomatic intracranial hemorrhage increases poor prognosis and mortality (30–32). The underlying mechanism is likely related to the disruption of the blood–brain barrier (BBB) within the first hour after ischemia. Ischemia-induced endothelial damage and increased vascular permeability can lead to hemorrhagic transformation following reperfusion therapy (33, 34). This study found that poor long-term outcomes in both the modeling and validation groups were associated with symptomatic hemorrhage, which is consistent with previous research. This highlights the importance of analyzing related factors of symptomatic intracranial hemorrhage and actively controlling relevant risk factors to reduce or prevent poor prognoses.

This study demonstrates that good collateral circulation serves as a protective factor against poor outcomes at 3 months. It is widely accepted that collateral circulation plays an irreplaceable role in maintaining blood flow reperfusion in ischemic areas. Good collateral circulation helps sustain blood supply to the ischemic penumbra, slows infarct volume growth, delays neurological damage, and produces retrograde perfusion, maximizing the rescue of the ischemic penumbra. It is a protective factor for long-term postoperative prognosis (35–37).

In our model, the time from puncture to reperfusion was shown to be an independent risk factor for poor long-term prognosis, and it is the only controllable factor. Previous studies have shown that the time from puncture to vascular recanalization is an independent risk factor for poor long-term outcomes after mechanical thrombectomy (9). During model development, we found that reperfusion time and symptomatic hemorrhage influenced by pre-procedural assessments, operator skill, device selection, and surgical strategy, collectively determine the success of the procedure, its duration, and the likelihood of hemorrhage, all of which significantly impact prognosis. The establishment of this model serves as a reminder to physicians to refine their techniques, optimize equipment choices, and enhance thrombectomy skills and procedural flow, ultimately improving long-term patient outcomes.

As age increases, the number of comorbidities such as atrial fibrillation, coronary heart disease, hypertension, and diabetes also increases in stroke patients. Elderly patients often have poor brain tissue and vascular conditions, as well as reduced physical function, leading to significant neurological damage at the time of onset, severe clinical symptoms, an increased risk of symptomatic hemorrhage after endovascular treatment, and difficulties in neurological recovery during rehabilitation, which ultimately results in poor long-term prognosis. Previous studies have shown that age is an independent risk factor for poor long-term outcomes after surgery (17), with elderly patients having poorer outcomes and higher mortality at 3 months post-operation (9).

In conclusion, this study identified five factors—baseline NIHSS score, symptomatic hemorrhage, time from puncture to reperfusion, collateral circulation, and age—through Lasso regression, and established a nomogram model. This model has good predictive value and clinical utility, providing a decision-making reference for future clinical work. However, this study has certain limitations. One limitation of our study is the preselection of variables using univariate analysis before applying LASSO regression. While this approach has been used in previous studies, it may introduce selection bias and contradicts LASSO’s ability to handle high-dimensional data without prior filtering. Future research should evaluate whether applying LASSO directly to the entire dataset yields comparable or improved model performance. It is a retrospective study with a small sample size, which may lead to selection bias. The functional prognosis of patients with severe acute ischemic stroke is influenced not only by clinical and procedural factors but also by patient adherence to rehabilitation, socioeconomic conditions, and access to post-stroke care. This study did not explore these potential contributors, highlighting an area for future investigation. Given the long study period, advancements in EVT techniques and improvements in physician expertise may alter the predictive factors associated with poor long-term prognosis. Future studies should consider evaluating how evolving treatment strategies impact prognostic modeling. Further research is needed to validate our predictive model in larger, multicenter cohorts to assess its applicability across diverse populations and healthcare settings. Incorporating additional biomarkers, imaging-based features, and artificial intelligence-driven approaches may further enhance the accuracy and clinical utility of prognostic models in the future.

## Data Availability

The original contributions presented in the study are included in the article/supplementary material, further inquiries can be directed to the corresponding author.
